# Design, Fabrication, and Dynamic Environmental Test of a Piezoresistive Pressure Sensor

**DOI:** 10.3390/mi13071142

**Published:** 2022-07-19

**Authors:** Rui Gao, Wenjun Zhang, Junmin Jing, Zhiwei Liao, Zhou Zhao, Bin Yao, Huiyu Zhang, Yuzhen Guo, Yanbo Xu, Yonghua Wang, Zengxing Zhang, Zhidong Zhang, Chenyang Xue

**Affiliations:** 1 State Key Laboratory of Dynamic Measurement Technology, North University of China, Taiyuan 030051, China; 18406583750@163.com (R.G.); junmin-jing@outlook.com (J.J.); s2006174@st.nuc.edu.cn (Z.L.); Zzzzhou95@163.com (Z.Z.); yao_bin2021@163.com (B.Y.); zshy980828@163.com (H.Z.); gyz20000113@163.com (Y.G.); xuyanbo_2001@163.com (Y.X.); wangyonghua@nuc.edu.cn (Y.W.); xuechenyang@nuc.edu.cn (C.X.); 2 School of Aerospace Engineering, Xiamen University, Xiamen 361102, China; 13485461170@163.com

**Keywords:** piezoresistive pressure sensor, silicon-on-insulator (SOI) structure, dynamic environment test

## Abstract

Microelectromechanical system (MEMS) pressure sensors have a wide range of applications based on the advantages of mature technology and easy integration. Among them, piezoresistive sensors have attracted great attention with the advantage of simple back-end processing circuits. However, less research has been reported on the performance of piezoresistive pressure sensors in dynamic environments, especially considering the vibrations and shocks frequently encountered during the application of the sensors. To address these issues, this paper proposes a design method for a MEMS piezoresistive pressure sensor, and the fabricated sensor is evaluated in a series of systematic dynamic environmental adaptability tests. After testing, the output sensitivity of the sensor chip was 9.21 mV∙bar^−1^, while the nonlinearity was 0.069% FSS. The sensor overreacts to rapidly changing pressure environments and can withstand acceleration shocks of up to 20× *g*. In addition, the sensor is capable of providing normal output over the vibration frequency range of 0–5000 Hz with a temperature coefficient sensitivity of −0.30% FSS °C^−1^ over the temperature range of 0–80 °C. Our proposed sensor can play a key role in applications with wide pressure ranges, high-frequency vibrations, and high acceleration shocks, as well as guide MEMS-based pressure sensors in high pressure ranges and complex environmental adaptability in their design.

## 1. Introduction

Microelectromechanical system (MEMS)-based piezoresistive pressure sensors have wide applications in industrial construction [1,2,3,4], healthcare monitoring [5,6,7,8,9,10,11], and ocean depth detection due to their high process maturity, easy sensor integration, and easy signal processing [12,13]. However, systematic research of the sensor performance in harsh environments is lacking, although many studies have been reported on the design and manufacturing of such piezoresistive pressure sensors. Various interference factors such as temperature, vibration, shock, and strong light irradiation may cause the abnormal output of the sensitive unit of the piezoresistive pressure sensor when in practical application.

Enabled by smart-cut and other technologies, silicon-on-insulator (SOI) wafers have been made commercially available for CMOS (Complementary Metal Oxide Semiconductor) and MEMS. In addition to its significant advantages in manufacturing high-performance CMOS circuits, SOI has been demonstrated as a perfect option for MEMS fabrication, owing to its excellent electrical isolation. Thanks to SOI technology, the pressure sensor’s fabrication process is becoming easier, and the sensor’s temperature preference can be enhanced significantly.

The sensor materials play an important role in enhancing the stability of the MEMS pressure sensor in a harsh environment. The drift of carriers in silicon material makes the piezoresistive pressure sensor unstable in high-temperature environments. In 2015, Sainan Li et al. achieved stable operation of a silicon-based piezoresistive pressure sensor at 350 °C by using the method of accurately calculating the heavy doping dose [14]. However, due to the inherently narrow forbidden band of silicon materials, pressure testing above 300 °C is still a difficult problem for silicon-based piezoresistive pressure sensors. Therefore, the breakthrough of high-temperature-resistant materials has become a research hotspot. Over the years, sensitive materials for piezoresistive pressure sensors have progressed, and silicon carbide (SiC) corresponds to one of the current candidates. Notably, SiC is one of the most promising materials for applications in harsh environments, thanks to its excellent electrical, mechanical, and chemical properties [15,16].

**Figure 1:**
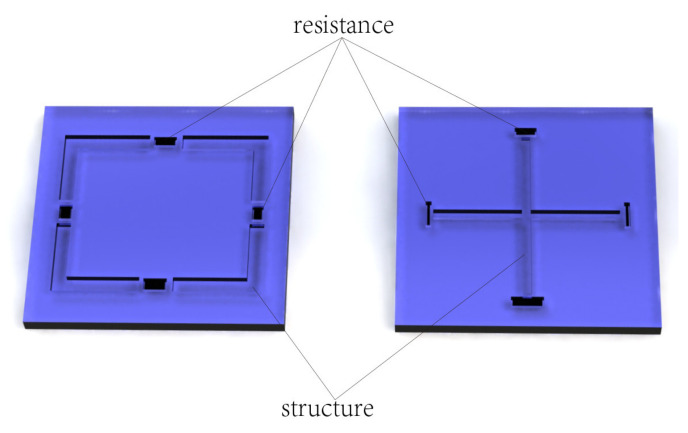
Etching a groove on the film structure to improve sensitivity might lead to instability in a harsh environment.

The sensor structure also plays an imperative role in sensor performance in harsh environments [17,18,19]. Various new film structures have been developed for increasing the sensor sensitivity while maintaining linearity, such as E-type film [20], hollow enhanced film [21], circular groove film [22], and peninsula structure film [23]. Using the optimized design of the diaphragm structure [20,21,22,23], the sensitivity of the sensor indeed increases, compared with the traditional C-type structure. However, as shown in Figure 1, etching grooves on the upper surface of the pressure-bearing diaphragm or adding a beam structure to the lower surface of the diaphragm will certainly have an impact on the natural frequency of the sensor. In practical applications of the sensor, it may experience high-frequency and low-frequency vibrations, as well as high-acceleration shocks, suggesting that it cannot be employed in a harsh environment.

Due to the piezoresistive effect of the semiconductor, its resistance changes in the stressed state. Subsequently, the resistance change in the stress concentration area of the diaphragm is sensed using the Wheatstone bridge circuit, which then converts the resistance change into a voltage change.

This study proposes a piezoresistive pressure sensor on an SOI substrate, which adopts the traditional C-type film structure, with a design range of 0–40 bar. The optimal parameters of the film structure are extracted through extensive finite element simulations. In addition, the relationship among the film size L, maximum stress size S, and film thickness h was established using a linear fitting method. To validate the design, the designed sensor chip was fabricated, and the sensor’s performance was tested in a harsh environment using a series of dynamic environmental tests including (a) a rapid pressure shock test, (b) a temperature test, (c) a high-frequency vibration test, and (d) a high-acceleration shock test.

Following the testing, the full-scale output of the pressure sensor prepared at room temperature reached 368.8 mV, while the temperature coefficient sensitivity was −0.3% FSS/°C. Furthermore, the dynamic environment test showed that the sensor could maintain a low output drift under 5000 Hz high-frequency vibration and 20× *g* acceleration shock. Our proposed sensor can play an important role in pressure measurement under high-frequency vibration and a high-acceleration shock environment, as well as guide the design of MEMS-based pressure sensors with high environmental adaptability. The performance comparison of the fabricated sensors is shown in the Table 1.

## 2. Structure Design and Simulation

**Figure 2:**
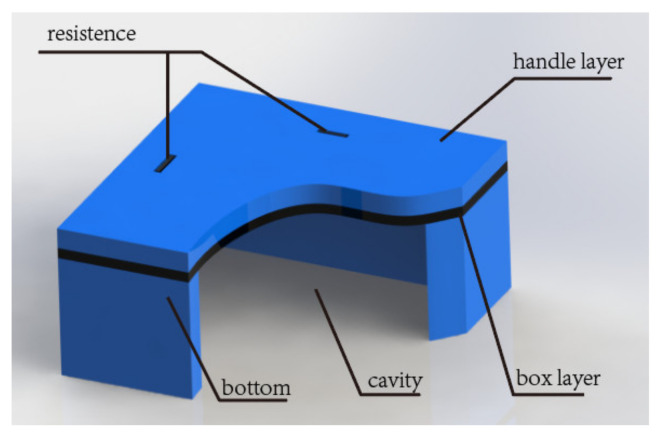
Diagram of the piezoresistive pressure sensor with back cavity. The thickness of the device layer is the sum of the thickness of the handle layer and the thickness of the box layer.

This work designs an absolute pressure sensor with a sensing range of 0–40 bar using a typical C-type film structure. The structural parameters are further optimized by the small deflection theory and finite element analysis. Due to the sandwiched structure of the SOI substrate, the fabrication of the sensitive diaphragm usually employs a buried oxide layer as an etch stop layer for silicon etching. The thicknesses of the buried oxide layer and device layer in the SOI substrate determine the thickness of the sensitive diaphragm, as shown in Figure 2.

**Figure 3:**
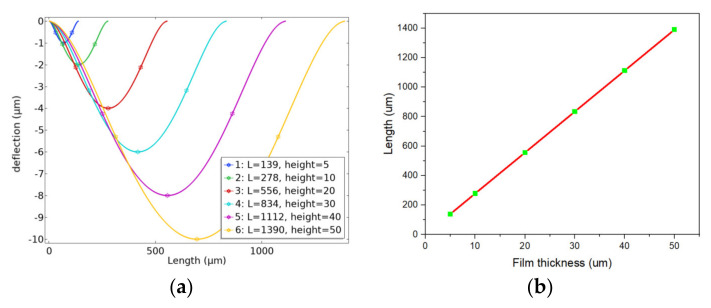
(**a**) Optimum film size for different film thicknesses; (**b**) relationship between film thickness and optimal film length.

We selected a diaphragm with various thicknesses to ensure that the size value reached the optimal linear stress under the maximum pressure. To find the optimal solution, the finite element analysis was used, which took the square film as the structure in the solid mechanics simulation. Film thicknesses of *h* = 5, 10, 20, 30, 40, and 50 µm were established. Next, the small deflection theory was used as the boundary condition to simulate and calculate the diaphragm size with a deflection of 1/5 h [26,27], under the maximum pressure. The finite element simulation results are shown in Figure 3a. Meanwhile, the relationship between film thickness and optimal film length is demonstrated in Figure 3b. It can be seen from Figure 3b that the optimal film length is proportional to the film thickness, and the fitting equation is given as follows:

*L* = 27.8 *h*. (1)

**Figure 4:**
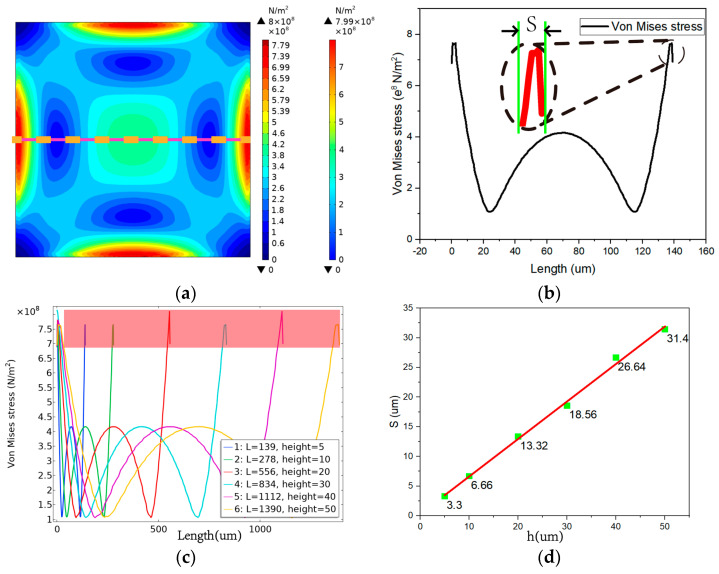
(**a**) Sensitive diaphragm stress distribution; (**b**) optimal stress length *S*; (**c**) centerline stress distribution; (**d**) relationship between the thickness of the sensitive diaphragm and the length S of the stress region.

To effectively sense the stress-induced resistant change in the piezoresistive diaphragm, the Wheatstone bridge consisting of four resistors should be placed as close as possible in the stress-concentrated area. The stress distribution of the diaphragm is shown in Figure 4a. Moreover, the centerline stress distribution and the maximum length of the stress concentration area S of the diaphragm are shown in Figure 4b. The maximum stress σ_max_ on the diaphragm is near the edge, starting from the edge of the diaphragm and moving inward along the centerline until the second point of equal stress is encountered. The distance S between the two points can be used as a reference for the design dimensions of the sensor resistance. *S* should be the focus when laying out the Wheatstone bridge and sizing the sensitive resistors, i.e., the optimum stress length.

Different optimal film sizes, the sensitive film under maximum pressure, and the centerline stress distribution are shown in Figure 4c. The maximum stress values of the diaphragms with different optimal sizes tended to be consistent under full-scale pressure. Evidently, the optimal stress region length S increased with the size of the diaphragm, and the variation trend of S with the film thickness is illustrated in Figure 4d.

The finite element simulation results revealed that S increased with the increase in film thickness *h*, with a large value of S indicating a larger space for the arrangement of resistors. This actually indicates that, in the design process, the thickness of the sensor sensitive diaphragm should be appropriately increased, and the fitting equation of *S* and film thickness *h* can be given as follows:

*S* = 0.632329 *h* + 0.311507. (2)

The thickness of the designed sensitive diaphragm in this work was 11 µm. Calculated according to Equation (1), the side length of the square diaphragm was L = 305.8 µm, while the optimum stress length calculated using Equation (2) was *S* = 7.267 µm. The structural parameters of the sensor are shown in Table 2.

## 3. Fabrication and Measurement

**Figure 5:**
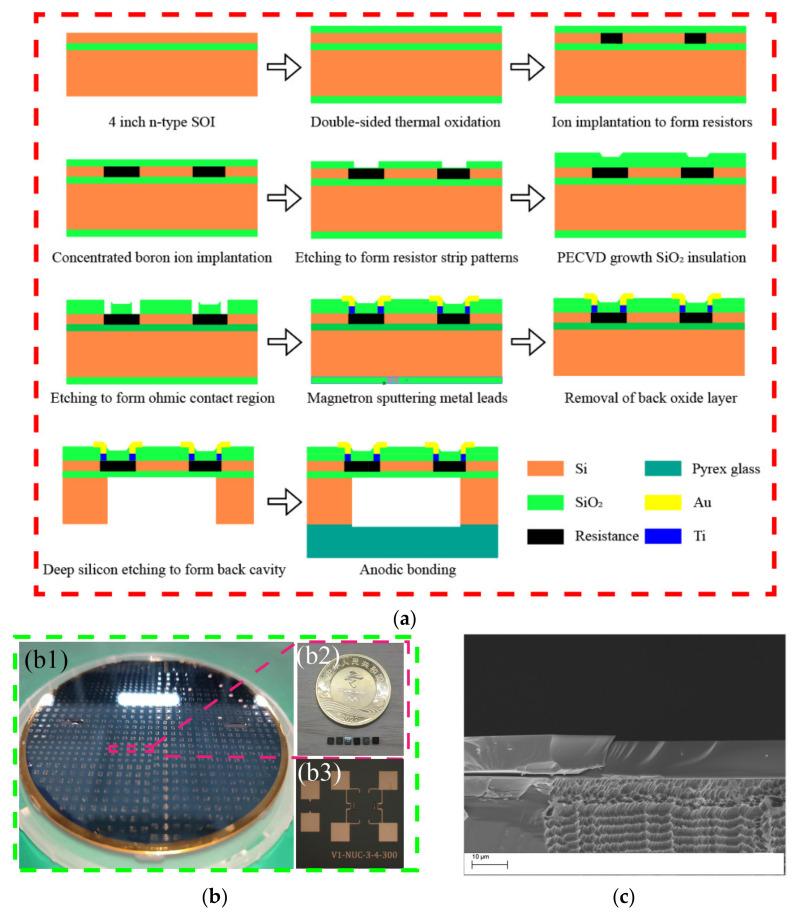
(**a**) Diagram illustrating chip manufacturing process; (**b1**) diagram of fabricated sensor wafers; (**b2**) diagram of several individual pressure sensor chips; (**b3**) diagram of the top surface of the chip under the microscope; (**c**) diagram of cross-section of chip under SEM (scanning electron microscope).

The cross-sectional schematic diagrams of the fabrication process for the pressure sensor are shown in Figure 5a. A double-side polished, 400 µm ± 5 um thick, 4 inch (100 ± 0.5 mm) P-type <100> SOI wafer with a resistance of more than 10,000 Ω·cm was used as the substrate, where the thicknesses of the handle layer and buried oxide layer were 10 µm and 1 µm, respectively. The fabrication process is summarized below. 

Initially, silicon dioxide (SiO_2_) was grown on both sides of the SOI wafer by thermal oxidation, which served as the protection layer. Then, lithography was performed on the front side of SOI substrate to pattern the area for piezoresistors, followed by boron ion implantation to form the piezoresistors. Next, the ohmic contact area at the end of the resistive strip was patterned by photolithography, and a high-concentration boron ion implantation was performed on the front side of the SOI wafer, to form a highly doped region near the sensitive cell. Then, the SOI wafer was rapidly annealed at 1000 °C for 20 s to activate the impurities. Later, SiO_2_ deposited by PECVD (plasma-enhanced chemical vapor deposition) was adapted as a passivation layer to protect the piezoresistor, while the ohmic contact region was exposed by wet etching of SiO_2_. Then, Ti/Au bonding pads were patterned to connect the resistors, to form a Wheatstone bridge. Finally, the Bosch etching process and anodic bonding created a vacuum back cavity to form an absolute pressure sensor [28,29]. An appropriate etching rate can ensure the verticality during the etching process to ensure the accuracy of the diaphragm size (Bosch etching rate of 1.6 µm per minute). The fabricated sensor chip was observed using SEM (scanning electron microscope), as shown in Figure 5c. The high verticality of the rear cavity ensured good diaphragm consistency and a good stress response. The actual chip manufactured in this work is shown in Figure 5b, indicating that the manufacturing was in line with the design expectations.

**Figure 6:**
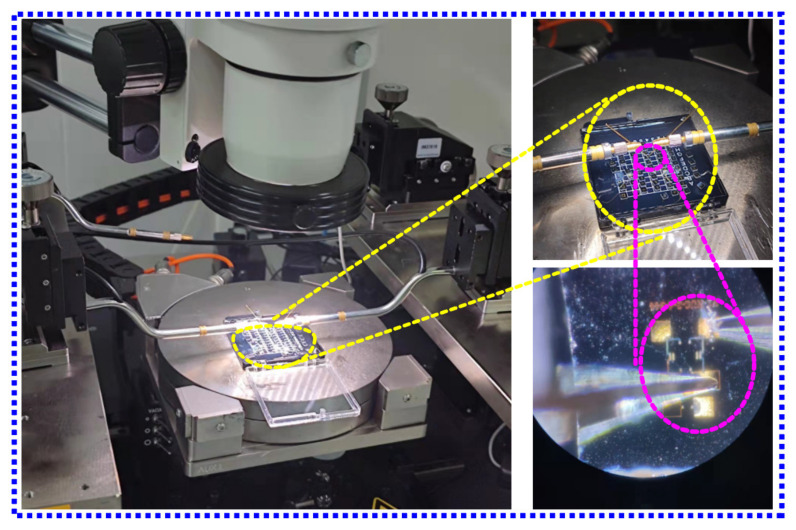
Diagram of semiconductor analyzer and a four-probe table.

**Figure 7:**
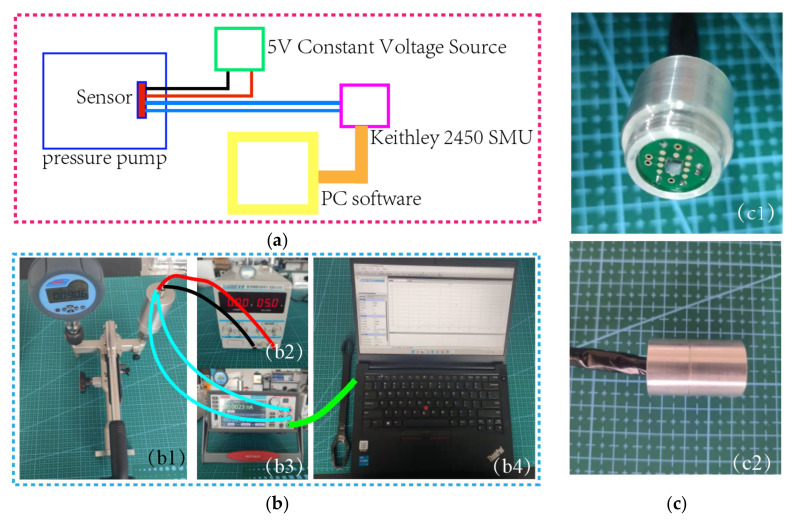
(**a**) Diagram of basic test process; (**b1**) diagram of pressure pump; (**b2**) diagram of DC stabilized power supply; (**b3**) Keithley 2450 SourceMeter SMU; (**b4**) diagram of PC software; (**c1**) diagram of axonometric view of packaged sensor; (**c2**) side view of packaged sensor.

The basic testing process of the sensor is described below. A semiconductor analyzer and a four-probe table were utilized to test the bridge resistance, as shown in Figure 6. A standard 6 MPa pressure test pump (Const 118) was used to pressurize the sensor, and the minimum control unit of the pump was 0.1 kPa, which could accurately control the pressure. A constant 5 V voltage source was used to power the sensor, and the output voltage signal of the sensor was monitored using a Keithley 2450 SourceMeter SMU. During the test, the pressure was changed with a step of 4.75 bar, and a total of nine points were selected. The pressure applied to the sensor was increased to a high value of 40 bar, and then gradually reduced from 40 bar to 2 bar. At each transition point, data were recorded only after the sensor output was stable, and then the basic index of the sensor was calculated. The basic test procedure of the chip is elaborated in Figure 7a,b.

The packaged chip is shown in Figure 7c. As shown in the figure, aluminum alloy was used as the shell of the sensor package, and the back of the sensor was potted with epoxy resin. In addition, an aluminum alloy protective cover was added to the front end of the package shell to protect the chip.

We also designed a series of experiments to verify the responsiveness and reliability of the fabricated pressure sensor while facing complex environments. Pressure is a continuous curve over time, and, when the sensor is used in a complex environment, it will encounter sudden changes in temperature, pressure, vibration, acceleration shock, etc. Likewise, these actual conditions can bring significant challenges to the sensor chip. The question is whether the sensor can respond correctly to these rapidly changing environmental conditions. In view of the above problems, the designed experiments were as follows: (a) six reference points were selected from 10 bar to 35 bar (10 bar, 15 bar, 20 bar, 25 bar, 30 bar, and 35 bar), and a pressure shock was applied at each reference point to conduct a rapid response test of the sensor; (b) a temperature experiment was conducted on the sensor, and a pressure scan was performed with the sensor at different temperatures; (c) the sensor was placed on the vibration table, with the sensor chip film perpendicular to the vibration direction, 10–5000 Hz frequency vibration was applied to the sensor, and the sensor output was observed; (d) 5×, 10×, 15×, and 20× *g* gravitational acceleration shocks were applied to the sensor, and the experiment was repeated five times to verify the reliability of the sensor.

## 4. Results and Discussion

The sensor chip was tested by a semiconductor analyzer, where it was found that the sensor units on the edge of the SOI chip possessed an abnormal Wheatstone bridge resistance, and the volt–ampere characteristic curve showed nonlinearity, which may have been due to the uneven implantation of boron ions making Schottky contacts in some areas of the SOI wafer; subsequently, the sensor chip with a resistance value closest to the design value was selected as the test sample.

**Figure 8:**
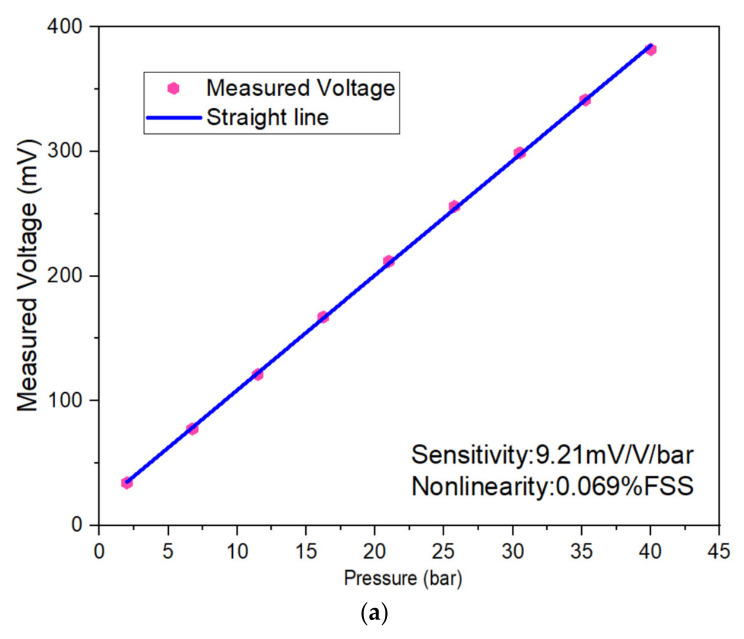

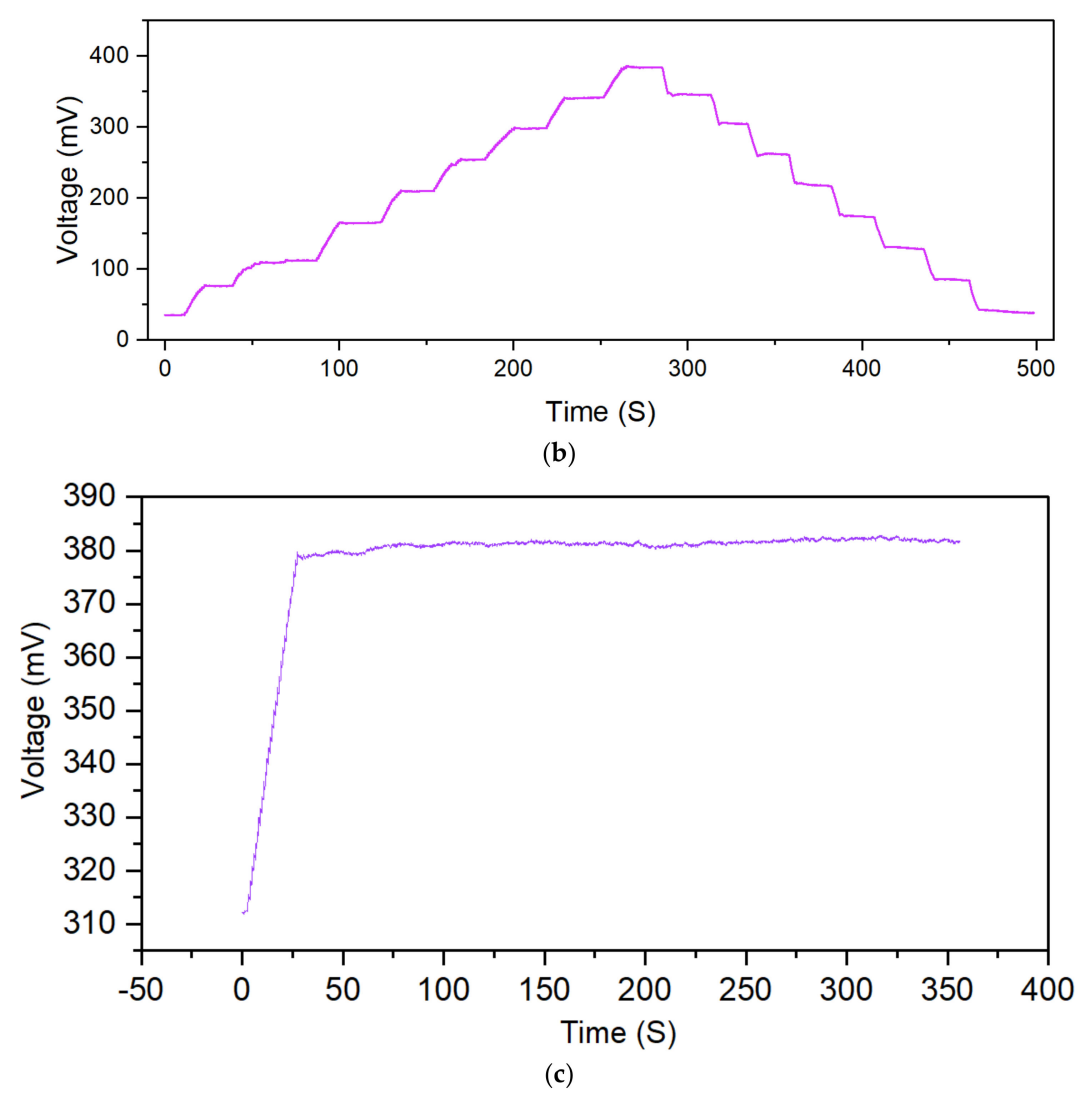
(**a**) Output voltage of the sensor; (**b**) forward and reverse step pressure test; (**c**) diagram of the output of sensor chip at full-scale pressure.

The measurement indicators of the sensor included zero output, sensitivity, nonlinearity, hysteresis, and maximum overload. The sensor’s full-scale pressure scan and sensor nonlinearity are shown in Figure 8a, while the positive and negative stroke pressure scans of the sensor are shown in Figure 8b. First, the sensor was kept under a full-scale pressure environment for 5 min, and the corresponding output of the sensor chip is shown in Figure 8c. Next, the sensor’s nonlinearity, repeatability, temperature coefficient sensitivity (TCS), and hysteresis were calculated [30]. The detailed data of the sensor at room temperature are shown in Table 3. The sensor had a sensitivity of 9.21 mV/V/bar and a nonlinear error of 0.069% FSS.

**Figure 9:**
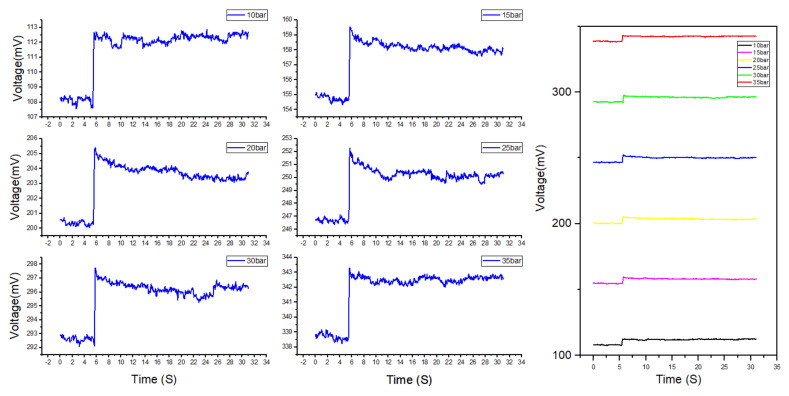
Diagram of sensor output during the rapid shock; the left picture is the separate output of the six sampling points, and the right picture shows a comparison of the six-point output. All pressure shocks were 0.4 bar.

To validate the dynamic performance and environmental stability of the sensor, a dynamic environmental adaptability test of the sensor was carried out. First, the sensor was tested for a rapid pressure increase. The test results under a rapid pressure increase are shown in Figure 9. Pressure values of 10, 15, 20, 25, 30, and 35 bar were selected as the base points, and a 0.4 bar instantaneous shock was applied to the sensor within 180 ms. The growth rate of the pressure shock was 2.22 bar/s. When the pressure sensor was impacted, the sensor’s output had a prominent peak, and it subsequently tended to be stable.

The shock was 0.4 bar, and the response time of the pressure pump was 10 ms. The difference between the voltage peak output of the sensor and the average value is shown in Table 4 Evidently, the maximum instantaneous shock error was 0.53% FSS. It can be seen from Figure 8 that the rapid increase in pressure caused the sensor to have a peak error. However, 2 s after the output peaked, the sensor output tended to a stable value, and the sensor’s error after stabilization reached 0.27%. Therefore, we can conclude that, when the pressure changes rapidly, the sensor will output excessive voltage, and the sensor output spike errors must be considered.

**Figure 10:**
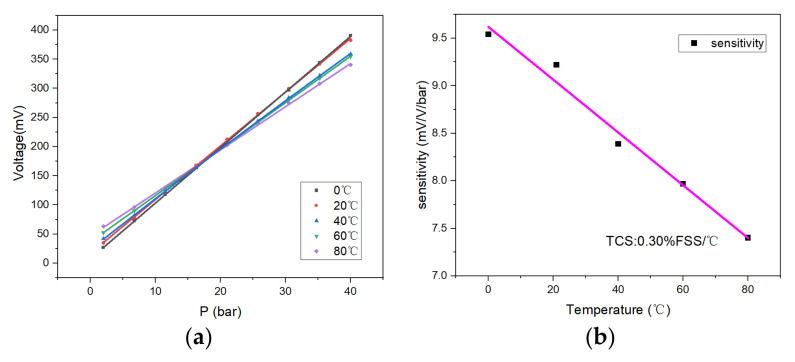
(**a**) Output of the sensor at different temperatures; (**b**) sensitivity drift at different temperatures.

Figure 10a indicates the sensor’s output voltage for temperature ranging from 0 to 80 °C. As the temperature increased, the zero-point output voltage of the sensor increased, and the slope of fitting curve of the sensor decreased, indicating that the sensitivity of the sensor diminished with the growth of the temperature. Here, the data fitting for the sensor sensitivity was carried out at different temperatures. Taking the sensor sensitivity at 20 °C as the benchmark, the temperature sensitivity coefficient (TCS) of the sensor was −0.03% FSS/°C. The sensitivity of the sensor dropped by 19.6% at 80 °C compared to the output at 20 °C. Nevertheless, the minimum resolution of the sensor could still reach 0.337% FSS. Moreover, the chip’s sensitivity at different temperatures is shown in Figure 10b.

**Figure 11:**
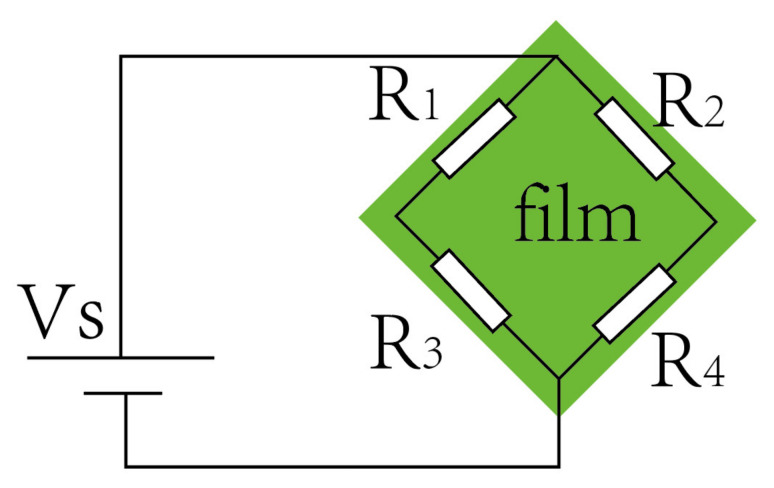
Schematic diagram of the Wheatstone bridge circuit.

It can be observed that, as the temperature increased, the sensitivity of the sensor decreased, while the zero-output increased (for the convenience of comparison, this paper regards the output of the pressure sensor as the zero output at 2 bar). This is because the carrier mobility of silicon decreases with the increasing temperature; thus, the piezoresistive coefficient of the sensor decreases [31], resulting in a decrease in the sensor sensitivity. The ideal sensor chip has a uniform doping concentration and four identical resistors (R1 = R2 = R3 = R4). As shown in Figure 11, according to the voltage output formula of the Wheatstone bridge [32], the zero output of the sensor chip should be 0 mV; however, the output of the fabricated sensor chip was 16.5 mV. As the temperature increases, the ratio of ΔA to ΔB changes; hence, the zero output of the sensor changes. This phenomenon might be caused by various reasons, among which the consistency of bridge resistance and the residual stress of the chip are two critical influencing factors. The boron ion doping error can also easily produce a resistance bar error. Under the supply voltage of 5 V, a resistance difference of 10 Ω can lead to a zero-point output drift of 13.1 mV. Notably, the SiO_2_ insulating layer may also cause this phenomenon after high-temperature annealing at 1000 °C, due to residual tensile stress [33,34,35].

(3) $$V_{\mathrm{out}}=V_{\mathrm{in}}\times \frac{R2\times R4-R3\times R1}{\left(R1+R2\right)\left(R3+R4\right)}.$$

(4) A=R2×R4−R3×R1.

(5) $$B=\left(R1+R2\right)\left(R3+R4\right).$$

**Figure 12:**
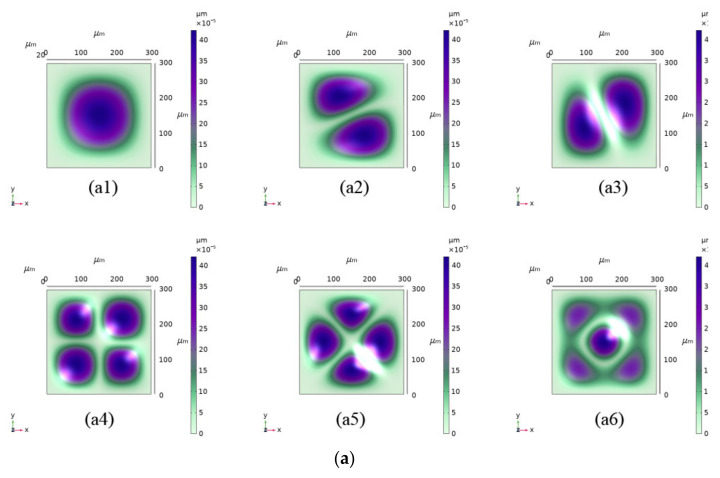

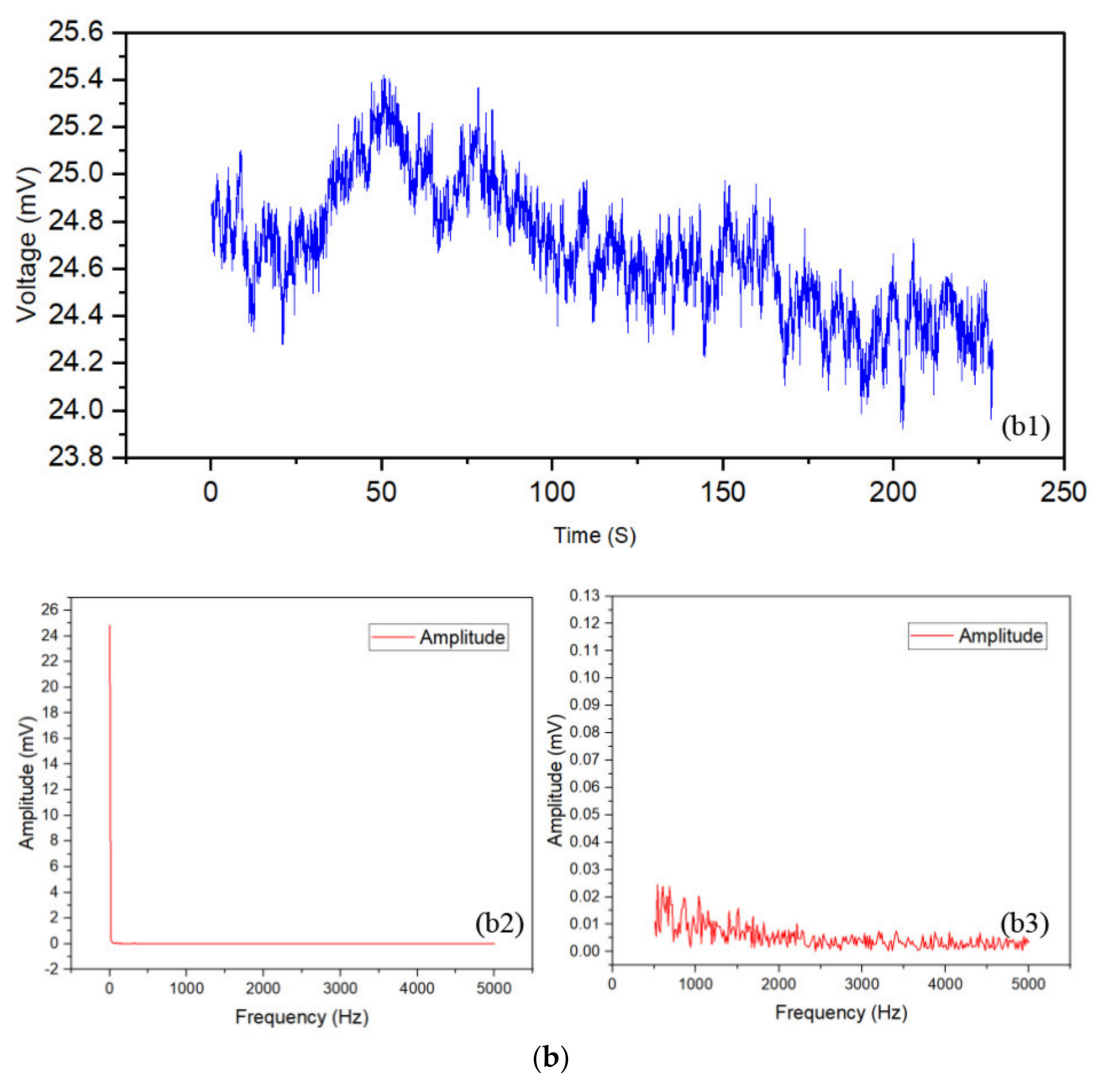
(**a**) Sensor eigenfrequency simulation. The diaphragm had six eigenfrequencies corresponding to six vibration modes: (**a1**) 1,777,100 Hz; (**a2**) 3,584,700 Hz; (**a3**) 3,583,800 Hz; (**a4**) 5,232,400 Hz; (**a5**) 6,328,600 Hz; (**a6**) 6,364,100 Hz. (**b1**) Output during the vibration sweep; (**b2**) Fourier-transform spectrogram based on b1; (**b3**) frequency-domain characteristics of sensor output during 500–5000 Hz vibration, the max amplitude is 0.025 mV.

A finite element simulation of the characteristic frequency of the sensor was carried out, and the results are reported in Figure 12a. It is concluded that the smallest characteristic frequency of the sensor was 1,777,100 Hz [36] when the normal atmospheric pressure (92.5 kPa) was 20 °C. At ambient pressure (92.5 kPa) and temperature (20 °C), the sensor was subjected to 0–5000 Hz vibration scanning. When the direction of sensor-sensitive film was parallel to the vibration direction, the output during the vibration sweep was as shown in Figure 12(b1). The data were subjected to Fourier transform [37] to study the frequency-domain characteristics, as shown in Figure 12(b2). It can be observed that the zero-point output of the sensor was within the test range of 0–5000 Hz, and the sensor chip did not resonate, as expected by the simulation. The output drift of the sensor was 0.37% FSS, indicating that the sensor had a good anti-vibration performance, and that it can be applied in a high-frequency vibration environment. It is worth mentioning that, during the test, the package shell of the sensor caused resonance at the vibration frequency of 700–750 Hz; the short-term resonance did not cause any abnormal sensor output. However, long-term resonance is very likely to damage the chip; thus, in a high-frequency vibration environment, the natural frequency of the sensor package structure must be considered as one of the critical indicators in the package design.

**Figure 13:**
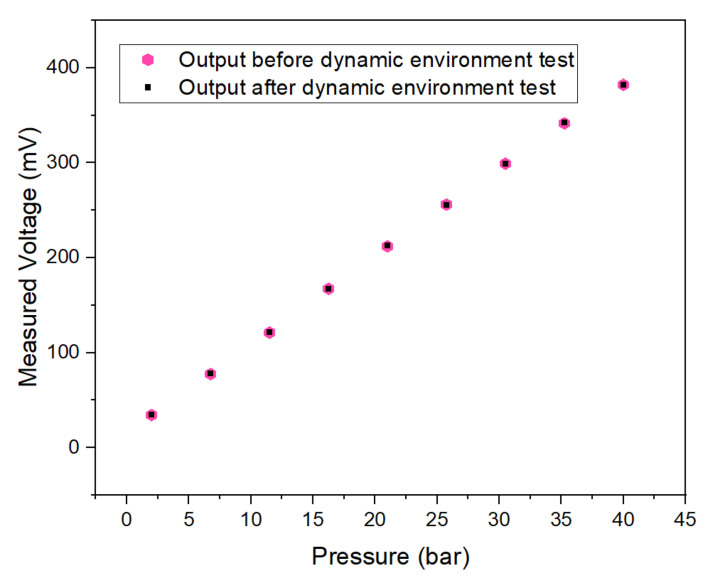
Sensor output before and after dynamic test.

After the sensor was tested by the above-simulated impact test, it was tested in the pressure pump. The result shows that the output of the sensor was normal, as shown in Figure 13.

## 5. Conclusions

This paper proposed an optimal design method for a C-structure piezoresistive pressure sensor and presented the optimal sensor parameter equation through finite element simulation. The designed sensor was manufactured and tested, and the test results validated the accuracy of the finite element simulation results. In addition, the fabricated sensor had high sensitivity and linearity, with a full-scale output of 368.8 mV and a nonlinear error of 0.069% over a pressure range of 40 bar. Meanwhile, the TCS of the sensor at 0–80 °C was −0.3% FSS/°C. Moreover, the sensor was capable of resisting a high-frequency vibration of 100–5000 Hz and an acceleration up to 20× *g*. In addition, according to the dynamic environment simulation test, we can conclude that the sensor’s output error became more significant when the sensor faced extremely rapid pressure pulses. It was also concluded that the anti-vibration performance of the sensor mainly depended on the packaging structure of the sensor. Therefore, to achieve a high sensor accuracy in a complex application environment, the influence of the environment should be one of the main reference factors in the design.

## 6. Patents

On the basis of this research, the team applied for a patent titled: An MEMS piezoresistive pressure sensor (Patent number: ZL 2021 2 2838774,X).

## Figures and Tables

**Table 1:** Comparison of performance with other pressure sensors at room temperature.

Sensor	Sensitivity (mV/V/bar)	Pressure Nonlinearity (%FSS)	Full Range Pressure
Proposed sensor	9.21	0.069%	40 bar
Sensor in [24]	6.03	3.89%	30 bar
Sensor in [24]	6.58	0.33%	10 bar
Sensor in [25]	5.18	0.02%	30 bar
Sensor in [25]	3.69	0.011%	30 bar

**Table 2:** Sizes of structural parameters of the sensor chip.

Parameter	Diaphragm Length	Diaphragm Thickness	Resistor Length	Resistor Width	Cavity Height	Chip Size
**Value (µm)**	306	11	80	8	389	2500*2500

**Table 3:** Technical data of the sensor at room temperature.

Parameter	Value	Parameter	Value
Resistance (kΩ)	1.9	Nonlinearity (%FSS)	0.069
Zero output (mV)	16.5	TCS (%FSS/°C)	−0.030
Sensitivity (mV/V/bar)	9.21	Full-range time drift (mV/V/min)	0.553
Repeatability (%FSS)	0.49	Hysteresis (%FSS)	2.6

**Table 4:** Sensor spike shock data.

Pressure/bar	10	15	20	25	30	35
**Average voltage (mV)**	112.25	158.21	203.80	250.30	296.27	342.51
**Peak voltage (mV)**	112.87	159.55	205.39	252.27	297.74	343.29
**Δ** **Peak voltage (mV)**	0.611	1.340	1.590	1.968	1.470	0.771

## Data Availability

Data are contained within the article.
